# Management of HIV Infection during Pregnancy in the United States: Updated Evidence-Based Recommendations and Future Potential Practices

**DOI:** 10.1155/2016/7594306

**Published:** 2016-07-18

**Authors:** Bassam H. Rimawi, Lisa Haddad, Martina L. Badell, Rana Chakraborty

**Affiliations:** ^1^School of Medicine, Department of Gynecology and Obstetrics, Division of Maternal Fetal Medicine and Reproductive Infectious Diseases, Emory University, 550 Peachtree Street, Atlanta, GA 30308, USA; ^2^School of Medicine, Department of Gynecology and Obstetrics, Division of Family Planning, Emory University, 550 Peachtree Street, Atlanta, GA 30308, USA; ^3^School of Medicine, Department of Gynecology and Obstetrics, Division of Maternal Fetal Medicine, Emory University, 550 Peachtree Street, Atlanta, GA 30308, USA; ^4^School of Medicine, Department of Pediatrics, Division of Infectious Diseases, Emory University, 2015 Uppergate Drive, NE, Atlanta, GA 30322, USA

## Abstract

All HIV-infected women contemplating pregnancy should initiate combination antiretroviral therapy (cART), with a goal to achieve a maternal serum HIV RNA viral load beneath the laboratory level of detection prior to conceiving, as well as throughout their pregnancy. Successfully identifying HIV infection during pregnancy through screening tests is essential in order to prevent* in utero* and intrapartum transmission of HIV. Perinatal HIV transmission can be less than 1% when effective cART, associated with virologic suppression of HIV, is given during the ante-, intra-, and postpartum periods. Perinatal HIV guidelines, developed by organizations such as the World Health Organization, American College of Obstetricians and Gynecologists, and the US Department of Health and Human Services, are constantly evolving, and hence the aim of our review is to provide a useful concise review for medical providers caring for HIV-infected pregnant women, summarizing the latest and current recommendations in the United States.

## 1. Introduction

Neonatal HIV infections are a result of transmission from a mother to her unborn fetus* in utero*, or during the intrapartum period, or postpartum secondary to breastfeeding [1]. In the US, perinatal transmission has been reduced to less than 1% in many states, reflecting implementation of key interventions during pregnancy, including initiating cART to suppress viral load beneath the level of detection and avoidance of breastfeeding during the postpartum period [1, 2]. Perinatal HIV guidelines in the US are constantly evolving. Here, we present a concise review outlining the latest perinatal recommendations, as well as potential future practices for medical providers caring for HIV-infected pregnant women.

## 2. Incidence of Perinatal HIV Transmission

Globally, without intervention, the cumulative* in utero, *intrapartum, and postpartum HIV transmission rate is approximately 35–40% [2]. In breastfeeding populations, postpartum HIV transmission through breastfeeding contributes about 40–45% of all mother-to-child transmissions (http://www.unaids.org/en/media/unaids/contentassets/documents/unaidspublication/2011/20110609_JC2137_Global-Plan-Elimination-HIV-Children_en.pdf). Maternal HIV viral load level is by far the most predictive factor for perinatal HIV transmission. Higher HIV viral loads correlate with a greater risk of perinatal transmission, although transmission can occur with any viral load, even when the systemic plasma viral load is beneath the level of detection [3]. Globally, the transmission rates for HIV can be reduced to less than 1% in pregnant women being compliant on their cART with virologic suppression and other perinatal recommendations [4, 5]. Without any intervention, this transmission rate is closer to 25% [2]. Among Hispanic/Latino and Caucasian women, the rates of HIV transmission have remained relatively stable in 2012 (<2% and 1%, resp.) [1, 2].

## 3. Preconception Counseling

All reproductively aged HIV-infected women should seek counseling prior to contemplating pregnancy, so that a detailed discussion on childbearing can be established. A key focus of discussions should include prevention of mother-to-child transmission (MTCT) of HIV, by initiation or continuation of an appropriately selected cART regimen [6], compliance with these medications during pregnancy and the postpartum period, and identifying potential barriers [7] that may affect postpartum retention in HIV care [8, 9]. The ability to successfully achieve maximal viral suppression before conception and throughout pregnancy is by far the most predictive means of achieving the lowest risk of potential MTCT [6]. Preconception counseling should be aimed at identifying women who may be victims of intimate partner violence, depression, and other psychological or psychiatric illnesses that may serve as barriers to prevention of MTCT and to treat and gain control of these conditions prior to contemplating pregnancy [10]. These strategies will not only result in safer conception and better compliance with cART but also result in better pregnancy outcomes [10].

## 4. HIV-Concordant and Discordant Couples

Consultation should include an expert in perinatal medicine and/or an experienced specialist who cares for HIV infected individuals [11]. With discordant couples that involve HIV-infected women, conception via timed artificial insemination is the safest option either via self-insemination or via intrauterine insemination [12]. When the discordant couple involves an HIV-infected male, the safest option for pregnancy is via insemination with donor HIV negative sperm. If the couple does not desire donor sperm, semen analysis is recommended prior to attempted pregnancy to avoid unnecessary exposure to genital fluid if semen abnormalities exist. Also sperm preparation techniques followed by intrauterine insemination or* in vitro* fertilization may reduce exposure risks [12].

## 5. Antepartum Care of HIV-Infected Women

Pitfalls and missed opportunities in healthcare delivery include failure of medical providers to screen pregnant women for HIV, ideally in the first trimester or at their first prenatal visit [7]. Transmission events are often documented in pregnant women who present with limited or no prenatal care, and those who decline HIV screening during pregnancy [13]. Pregnant women who have not previously had HIV screening and present to a labor and delivery suite in labor urgently require rapid HIV testing at the time of admission [14]. If a positive rapid HIV test is noted, and these patients are deemed to be in active labor or delivery is warranted for other obstetrical indications, then immediately implementing interventions to reduce intrapartum HIV transmission is recommended, which includes a combination of maternal administration of at least 3 hours of intravenous zidovudine therapy prior to a cesarean delivery and postnatal infant prophylaxis with a dual ARV combination regimen of zidovudine and nevirapine [15]. Awaiting laboratory confirmation should not delay these urgent interventions [11].

**Figure 1:**
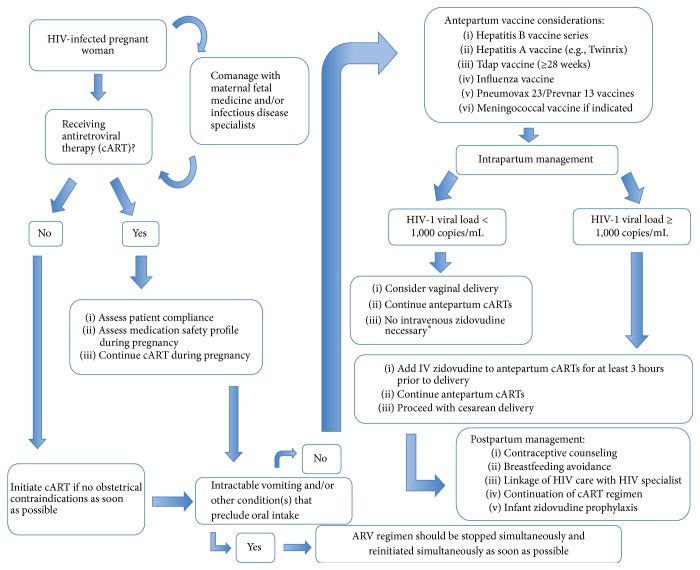
Algorithm for management of HIV during pregnancy. ^*∗*^Intravenous (IV) zidovudine is not required for HIV-infected women who are compliant with cART and who have an HIV-viral load < 1000 copies/mL at the time of delivery. HIV: human immunodeficiency virus, cART: combination antiretroviral therapy, PO: per os (by mouth), CBC: complete blood count, CMP: complete metabolic panel, CD4: cluster of differentiation 4, and mL: milliliter.

*For known positive mothers*, HIV RNA quantitative viral loads should be assessed monthly; however, some medical providers may consider wider interval blood draws to every 2 months, in those pregnant women who have consistently had a suppressed HIV-1 RNA viral load beneath the level of detection while on effective cART [16].  Figure 1 illustrates algorithmic management of HIV during pregnancy.

## 6. The Diagnosis of HIV during Pregnancy

A positive screening test with traditional HIV testing modalities, either with an HIV 1/2 Antigen/Antibody test or with a Fourth Generation test with reflexes essentially establishes a diagnosis of HIV infection [17]. Newer testing modalities include reflex Multispot rapid HIV testing, which includes a combined HIV-1/HIV-2 rapid test to distinguish between HIV-1 and 2 infection; hence, a positive Multispot test confirms a diagnosis of HIV [17, 18]. However, if the Multispot test is negative, additional reflex testing for establishing a diagnosis of HIV infection includes proceeding with a polymerase chain reaction (PCR) test [18]. The sensitivity and specificity of rapid HIV testing are close to 100%, while the positive predictive value (PPV) depends on the prevalence of the disease in the general population being tested [19, 20]. In populations in which the HIV prevalence is low, a lower PPV is noted [19].  Table 1 demonstrates the different diagnostic tests that are available for establishing a diagnosis of HIV in pregnant women.

## 7. Coinfection Screening and Vaccination Recommendations

In addition to routine prenatal labs obtained during the first trimester, or at entry into prenatal care, which already includes the assessment of hepatitis B and syphilis, screening for hepatitis C coinfection is recommended for HIV positive women [16]. Those who have a negative hepatitis B surface antibody status should receive hepatitis B vaccine series during pregnancy, regardless of which trimester they are in, as well as screening for immunity to hepatitis A, as a combination vaccine for hepatitis B and hepatitis A exists, formerly known as Twinrex [21]. Patients who are coinfected with these other viral entities should seek consultation with HIV and hepatitis-experienced medical provider(s). For patients with hepatitis B infection, all newborn infants should receive both the hepatitis B vaccine series and hepatitis B immune globulin preferably within 12 hours after delivery, regardless of the maternal hepatitis B viral load [22, 23].

In addition to vaccination against hepatitis A and B during pregnancy, additional vaccines to offer HIV-infected women during pregnancy should include the influenza vaccine (inactivated) during the influenza season, which can be offered to unvaccinated pregnant women during any trimester of pregnancy, as well as combined diphtheria, tetanus, and pertussis (Tdap) vaccine around 28–36 weeks of gestation [23]. Additional vaccines that may be given to HIV-infected pregnant women include Pneumovax 23 (23-valent pneumococcal polysaccharide) vaccine and Prevnar 13 (13-valent pneumococcal conjugate) vaccine [23]. Vaccines that should not be given during pregnancy and postponed until the postpartum period include vaccination against varicella, zoster, human papillomavirus (HPV) and measles, mumps, and rubella (MMR), if testing results are equivocal or nonimmune [23]. At this time, vaccination with serogroup B meningococcal (MenB) vaccine should not be given during pregnancy, as there is not enough information about the potential risks of this vaccine during pregnancy or during the postpartum period for breastfeeding women [24].

## 8. Safety of Antiretroviral Agents during Pregnancy

The most effective method of identifying adverse fetal/neonatal outcomes is to report all drug exposures to the Antiretroviral Pregnancy Registry [25]. The information needed to participate in this registry can be readily located online at http://www.apregistry.com/.  Table 2 illustrates the different treatment regimens by class for HIV-Infected pregnant women, while Table 3 illustrates the most common reported adverse pregnancy outcomes of certain ARVs.

Small cohort studies have raised the concern that ARVs during pregnancy were associated with low birth weight and preterm birth [26–29], while these concerns may be in fact due to the severity of the disease, rather than the association with ARVs [30]. Other studies have evaluated the use of protease inhibitors and the risk of preterm delivery (PTD) [31–34], as well as the use of zidovudine and the risk of congenital cardiac defects [35], while others have shown favorable pregnancy outcomes [36].

Specific ARV regimens from HIV-pregnant women who gave birth to HIV exposed HIV negative infants have been evaluated from a large cohort in sub-Sahara Africa of more than 3000 patients for their association with adverse pregnancy outcomes, specifically for PTD, small for gestational age (SGA), and low birth weight (LBW) [37, 38] in relation to the duration of ARV exposure. The authors noted a 30% increased risk of preterm delivery amongst infants ARV-exposed women, with the highest risk in women who initiated ARVs prior to conception, compared to those who initiated ARVs during pregnancy or received zidovudine monotherapy [37]. Similarly, there was a 20% increased risk of infants being diagnosed with SGA when exposed to ARVs during pregnancy and before conception; however, when they assessed different ARV exposures, there was no difference in the overall SGA neonates [37].

The highest rates of PTD (25%) and SGA (13%) were those women treated with protease inhibitors during pregnancy [37]. They also found an increased likelihood of LBW amongst women who initiated ARVs prior to conception and those who were exposed to ARVs during pregnancy. Overall, they found that pregnant women exposed to ARVs of longer duration with initiation of ARVs prior to pregnancy had the highest rates of PTD, SGA, and LBW [37]. It is unclear the exact mechanism of why these adverse pregnancy outcomes were seen [39, 40], and further comparative evaluations of different regimens are needed.

Intrapartum, maternal infusion of zidovudine dosing has been investigated and compared to therapeutic exposures in order to assess fetal concentrations [41]. Reducing the maternal infusion dosage from a loading dose of 2 mg/kg to 1 mg/kg over 1 hour, followed by a reduction in the maintenance dosage of 1 mg/kg to 0.5 mg/kg each hour until delivery, reduced fetal exposures [41]. This can also be achieved by taking zidovudine orally every 5 hours, starting at the onset of labor until the time of delivery, followed by neonatal zidovudine prophylaxis as soon as possible after birth [41]. This reduction in zidovudine dosage during the first few days of neonatal life also is important as well.

While treatment of HIV during pregnancy is crucial and the benefits outweigh the risks for the prevention of MTCT, the safety profile of ARVs during pregnancy has shown conflicting evidence. It is ideal that medical providers caring for HIV infected reproductive age women start ARVs prior to pregnancy and continue these throughout pregnancy, or as early as tolerated during pregnancy to achieve the ultimate goal of viral suppression and prevention of MTCT.

## 9. Postpartum Care

All HIV-infected women should have a routine postpartum follow-up visit with their obstetric provider. After delivery, establishing retention in HIV care is critical, with referral to an infectious disease specialist. Counseling patients regarding adherence to cART, irrespective of CD4 count, is very important, as some HIV-infected women become noncompliant with cART during follow-up after delivery.

Future efforts should be geared towards adapting new strategies in the US to achieve postpartum retention in HIV care settings [42], particularly because there are a limited number of available published reports addressing these strategies during the postpartum period. These strategies include discussion about unintended pregnancy prevention with contraceptive counseling, as well as HIV/STD preventive strategies such as consistent condom use and preexposure prophylaxis. In addition, similar to the preconception-counseling period, postpartum counseling should address intimate partner violence, postpartum depression, and other mental health issues that require treatment, as well as providing linkage to intimate partner violence services [43, 44].

## 10. Infant Follow-Up and Postnatal Prophylaxis

Regardless of maternal viral load, after birth, all babies born to HIV-infected women should be bathed immediately to remove any potentially infectious maternal secretions [18]. Baseline complete blood counts and HIV diagnostic testing by HIV DNA PCR are to establish or rule out HIV infection, followed by ZDV prophylaxis. Within 12 hours after birth, and ideally no later than 24 hours, all neonates born to HIV positive mothers should receive a course of ZDV therapy, which will be continued for 6 weeks in the US; however, a 4-week regimen can be considered for full-term infants whose mothers had maintained HIV viral suppression antenatally [45, 46].

A recent study evaluated three postpartum cART regimens for neonates born to mothers infected with HIV who have not received any antepartum cART or had a viral load of >1,000 copies/mL near the time of delivery [47]. Combination prophylaxis with either a 2-drug (zidovudine plus nevirapine) regimen or a 3-drug regimen (zidovudine plus nelfinavir and lamivudine) was found to have superior efficacy, when compared to zidovudine monotherapy alone, with significantly reduced transmission rates of HIV [47]. The Department of Health and Human Services (DHHS) for the US currently recommends that at least 3 doses of nevirapine within the first 7 days of life be administered, in addition to ZDV therapy as prophylaxis during high-risk perinatal exposure when maternal HIV viral load is or is assumed to be more than 1,000 copies/mL.

Premastication of food for infants of HIV-infected mothers should be avoided, as this can potentiate the risk of HIV transmission [48]. Free clinical perinatal HIV consultation, including the care of an HIV-exposed neonate, is available for medical providers at The National Perinatal HIV Hotline at 1–888-448-8765.

Although early-term deliveries can be associated with increased neonatal intensive care unit admissions and higher hospital costs, when compared to those beyond 39 weeks [49], the recommendations currently endorsed by the American College of Obstetricians and Gynecologists (ACOG) is for cesarean deliveries at 38 weeks for pregnant women with HIV-1 RNA viral loads > 1,000 copies/mL [50]. We currently recommend delivery prior to 39 weeks of gestation for other high-risk conditions if the benefit of earlier delivery outweighs the risk of expectant management; therefore, decreasing the risk of spontaneous labor/rupture of membranes between 38 to 39 weeks in women with an HIV-1 RNA viral loads > 1,000 copies/mL outweighs very small increased transient risks of delivery at 38 versus 39 weeks for the neonate [51].

## 11. Summary of Future Potential Practices

- It is ideal that medical providers caring for HIV infected reproductive age women start ARVs prior to pregnancy and continue these throughout pregnancy, or as early as tolerated during pregnancy to achieve the ultimate goal of viral suppression and prevention of MTCT.
- During the preconception counseling and postpartum periods, medical providers should identify women who may be victims of intimate partner violence, depression, and other psychological or psychiatric illnesses that may serve as barriers to prevention of MTCT and to treat and gain control of these conditions prior to contemplating pregnancy.
- These strategies not only will result in safer conception and better compliance with cART but also will result in better pregnancy outcomes and less adverse pregnancy outcomes, as these patients will be more compliant with their prenatal visits and follow recommendations outlined by their obstetrical provider.
- Prevention of pitfalls and missed opportunities in healthcare delivery can be achieved by medical providers screening all pregnant women for HIV, ideally in the first trimester or at their first prenatal visit, establishing an early diagnosis of HIV and initiating cART early in the pregnancy.
- Postpartum retention in HIV care settings is critical and can best be achieved through strategies that include a discussion about future unintended pregnancies with contraceptive counseling, as well as preventive strategies such as consistent condom use and preexposure prophylaxis.

## Figures and Tables

**Table 1:** Testing modalities for diagnosing HIV in pregnancy^*∗*^.

HIV tests	What they test for	Window period	Available results	Sensitivity	Specificity
ELISA	HIV antibodies	3 Months	2 days–2 weeks	>99%	>98%
Antigen test (p24)	P24 viral proteins	11 days–1 month	2 days–1 week	90%	100%
4th generation tests	Antibodies and p24	11 days–1 month	2 days–2 weeks	>99.7%	>99.3%
PCR/NAAT tests	Genetic material of HIV	12 days	2 days–1 week	>99%	>99%
Rapid test	Antibodies	3 Months	Within 20 minutes	>99%	>98%

^*∗*^AIDSinfo. Recommendations for use of antiretroviral drugs in pregnant HIV-1-infected women for maternal health and interventions to reduce perinatal HIV transmission in the United States. *HHS panel on treatment of HIV-infected pregnant women and prevention of perinatal transmission, a working group of the office of AIDS research advisory council (OARAC), *2015, http://aidsinfo.nih.gov/guidelines.

**Table 2:** Treatment regimens for HIV-infected pregnant women.

Brand name	Preparation	Comments
*Preferred regimens*		

*Two-NRTI backbone*		
Trizivir	ABC/3TC	Patients with an HIV RNA viral load > 100,000 copies/mL should not receive a combination therapy consisting of ABC/3TC with ATV/ritonavir or efavirenz.
Truvada	TDF/FTC or 3TC	TDF-based dual NRTI combinations should be used with caution in patients with renal insufficiency.
Combivir	ZDV/3TC	NRTI combination therapy requires twice daily administration and increases potential for hematologic toxicities.

*Protease inhibitor regimens*		
Reyataz	ATV/r plus a two-NRTI backbone	Maternal hyperbilirubinemia.
Prezista	DRV/r plus a two-NRTI backbone	Must be used twice daily in pregnancy.

*NNRTI regimen*		
Efavirenz	EFV plus a two-NRTI backbone^*∗*^	Concern because of birth defects seen in primate study, unclear risk in humans.

*Integrase inhibitor regimen*		
Raltegravir	RAL plus a two-NRTI backbone	Rapid viral load reduction. Twice-daily dosing required.

*Alternative regimens*		

*Protease inhibitor regimens*		
Kaletra	LPV/r	More nausea than preferred regimens. Twice-daily administration in pregnancy.

*NNRTI regimens*		
Complera	RPV/TDF/FTC (or RPV plus a two-NRTI backbone)	RPV not recommended with pretreatment HIV RNA > 100,000 copies/mL or CD4 cell count < 200 cells/mm^3^. Do not use with PPIs. PK data available in pregnancy but relatively little experience with use in pregnancy. Available in co formulated single-pill once daily regimen.

NRTI: nucleoside or nucleotide reverse transcriptase inhibitor, NNRTI: nonnucleoside or nonnucleotide reverse transcriptase inhibitor, ABC: abacavir, 3TC: lamivudine, TDF: tenofovir disoproxil, FTC: emtricitabine, ZDV: zidovudine, ATV: atazanavir, r: ritonavir (boosted regimen), DRV: darunavir, ^*∗*^EFV: efavirenz, recommended to be started after 8 weeks of gestation, RAL: raltegravir, LPV: lopinavir, and RPV: rilpivirine.

**Table 3:** Pregnancy outcomes of individual antiretroviral agents during pregnancy^*∗*^.

Brand name	Reported adverse pregnancy outcomes	
	Maternal	Fetal/neonatal
*NRTI*		
Zidovudine	Potential for hematologic toxicities (anemia and bone marrow suppression) [52], including elevated liver function tests [53], myelotoxicity [54], acute pancreatitis [54], preeclampsia, and other hypertensive disorders [55]	PTD [37], SGA [37], LBW [37], and CHD [35]
Tenofovir disoproxil fumarate	Kidney [56] and bone toxicity [57]	Decreased bone mineral density content [58]

*NNRTI*		
Efavirenz	Rash and drug interactions [59]	Initial concern of birth defects seen in primate study [60]; however, recent studies have not shown an increased risk of neural tube defects [61]
Abacavir	Abacavir should not be used in patients who test positive for HLA-B^*∗*^5701 because a positive test may increase the risk of a hypersensitivity reaction [56], nausea, vomiting, diarrhea, and abdominal pain [59]	None
Didanosine	Pancreatitis (acute and chronic) [54] and neuropathy	Initial studies concerning for an association with fetal anomalies, specifically head and neck anomalies when exposed during the first trimester [35]; however, recent studies found no adverse outcomes [62]
Nevirapine	10-fold increased risk of hepatotoxicity^¥^ [63, 64]	No reported fetal malformations [64]
Emtricitabine	Headache, nausea, vomiting, and diarrhea [59]	None [65]

*Protease inhibitors*		
Ritonavir	Nausea, vomiting, increased triglycerides, and transaminases [59]	
Atazanavir	Abdominal pain, diarrhea, nausea, and increased liver function tests [59]	PTD [31, 32, 34, 66]
Lopinavir	Nausea, vomiting, diarrhea, and pancreatitis [59]	PTD [21, 31, 34, 66]
Darunavir	Maternal hyperbilirubinemia and nausea [59]	PTD [31, 32, 34, 66]

^*∗*^Table provides a short list of the updated ARV's and their reported safety issues.

^*¥*^Nevirapine can cause fatal and severe hepatotoxicity among women with CD4 lymphocytes > 250 cells/*μ*L.

NRTI: nucleoside or nucleotide reverse transcriptase inhibitor, NNRTI: nonnucleoside or nonnucleotide reverse transcriptase inhibitor, PTD: preterm delivery (delivery prior to 37 weeks), SGA: small for gestational age (birth weight less than the 10th percentile for their gestational age), LBW: low birth weight (birth weight less than 2500 grams), and CHD: congenital heart defects.
